# Cultural variation in running techniques among non-industrial societies

**DOI:** 10.1017/ehs.2022.12

**Published:** 2022-04-11

**Authors:** Ian J. Wallace, Thomas S. Kraft, Vivek V. Venkataraman, Helen E. Davis, Nicholas B. Holowka, Alexandra R. Harris, Daniel E. Lieberman, Michael Gurven

**Affiliations:** 1 Department of Anthropology, University of New Mexico, Albuquerque, NM, USA,; 2 Department of Anthropology, University of Utah, Salt Lake City, UT, USA,; 3 Department of Anthropology and Archaeology, University of Calgary, Calgary, AB, Canada,; 4 Department of Human Evolutionary Biology, Harvard University, Cambridge, MA, USA,; 5 Department of Anthropology, University at Buffalo, Buffalo, NY, USA,; 6 Department of Archaeology and Anthropology, University of Cambridge, Cambridge, UK; 7 Department of Anthropology, University of California Santa Barbara, Santa Barbara, CA, USA

**Keywords:** Physical activity, kinematics, social learning, foot strike, overstride, Tsimane

## Abstract

Research among non-industrial societies suggests that body kinematics adopted during running vary between groups according to the cultural importance of running. Among groups in which running is common and an important part of cultural identity, runners tend to adopt what exercise scientists and coaches consider to be good technique for avoiding injury and maximising performance. In contrast, among groups in which running is not particularly culturally important, people tend to adopt suboptimal technique. This paper begins by describing key elements of good running technique, including landing with a forefoot or midfoot strike pattern and leg oriented roughly vertically. Next, we review evidence from non-industrial societies that cultural attitudes about running associate with variation in running techniques. Then, we present new data from Tsimane forager–horticulturalists in Bolivia. Our findings suggest that running is neither a common activity among the Tsimane nor is it considered an important part of cultural identity. We also demonstrate that when Tsimane do run, they tend to use suboptimal technique, specifically landing with a rearfoot strike pattern and leg protracted ahead of the knee (called overstriding). Finally, we discuss processes by which culture might influence variation in running techniques among non-industrial societies, including self-optimisation and social learning.

## Social media summary

New evidence from the Tsimane underscores that running techniques vary between societies according to the cultural importance of running

## Introduction

Culture influences how people move their bodies (Mauss, 1935), which is an idea of shared interest to researchers working in all fields of anthropology. Ethnographers, social anthropologists and linguistic anthropologists have studied how cultural norms and values affect patterns of body movement involved in dance, play, communication, rituals, food production, tool making and numerous other activities (e.g. Abel, 1984; Bateson, 1977; Bril, 1986; Farnell, 1995; Gandon et al., 2013; Hall, 1996; Kaeppler, 1985; Kendon, 1988; Mahias, 1993; Pelosse, 1981; Williams, 1975). Archaeological research often proceeds from the premise that material culture traditions are the result of manufacturing methods associated with body techniques that were socially learned and passed down through generations (e.g. Inizan et al., 1999; Lemonnier, 1986; Pétrequin, 1993; Roux, 2019; Tostevin, 2012). Biological anthropologists have emphasised how cultural, environmental and physiological factors interact in shaping variation in human body movement patterns (e.g. Holowka et al., in press; Kraft et al., 2014; Lieberman, 2014; Lieberman et al., 2010, 2015a; Venkataraman et al., 2012, 2018; Wallace et al., 2018a; Watanabe, 1971; Willems et al., 2017). Even primatologists have studied the ways that social learning shapes the gestures employed by our species’ closest living relatives – chimpanzees and bonobos – for grooming, communication and tool use (e.g. Boesh, 1991; Halina et al., 2013; Lonsdorf et al., 2004; Wrangham et al., 2016). Thus, achieving a better understanding of the ways that body movement patterns are shaped by culture can be considered a major goal of anthropology.

Notwithstanding interest among anthropologists, the influence of culture on the body techniques employed during many common types of physical activity has yet to be thoroughly explored. Here, we consider one such activity: running. More specifically, we consider running at submaximal speed (i.e. aerobic intensity), as opposed to sprinting. Over the past decade, evidence has accumulated from research among non-industrial societies that body movement patterns adopted during running tend to vary between groups according to the perceived cultural importance of running. Among groups that value running and consider it an important part of their cultural identity, people tend to adopt elements of what many exercise scientists and coaches consider to be good running technique for avoiding injury and maximising performance (Lieberman, 2014; Lieberman et al., 2010, 2015a). In contrast, among groups in which running is not considered important to cultural identity, people tend to adopt suboptimal techniques when they run (Hatala et al., 2013; Pontzer et al., 2014).

This paper is structured as follows: first, we briefly describe key differences between good and suboptimal running techniques. Second, we review recent evidence from non-industrial societies that cultural attitudes about running are associated with variation in running techniques. Third, we present new data on running frequency and technique collected among the Tsimane, a non-industrial forager–horticulturalist group living in the Bolivian Amazon. Our observations suggest that running is not a common activity among the Tsimane, and when Tsimane people do run, they tend to employ suboptimal running technique. Fourth and finally, we speculate about potential ways in which culture is responsible for shaping variation in running techniques among non-industrial societies.

### Good vs. suboptimal running techniques

Exercise scientists and coaches generally consider good running technique in terms of body kinematics that minimise susceptibility to injury and maximise performance (Anderson, 2018; Daniels, 2014; Lieberman, 2021). Few running-related injuries are caused by a single, traumatic event but instead by the cumulative effects of forces that act repeatedly on the body, often called repetitive stress injuries. Such injuries include plantar fasciitis, skeletal stress fractures, patellofemoral pain syndrome and iliotibial band syndrome. The forces that have shown the most consistent relationship with running-related repetitive stress injuries are impact transients (Davis et al., 2016; Johnson et al., 2020), the abrupt collision forces of approximately 1.5–3 times body weight that occur within the first 50 milliseconds after the foot strikes the ground. Thus, from the perspective of injury prevention, attenuation of impact transients is regarded as an important feature of good running technique (Lieberman, 2012, 2021; Lieberman et al., 2010).

Running performance is determined largely by three physiological factors: running economy, maximal oxygen uptake and blood lactate accumulation (Bassett & Howley, 2000; Conley & Krahenbuhl, 1980; Farrell et al., 1979; McLaughlin et al., 2010). Of these factors, the one most affected by running technique is running economy (Barnes & Kilding, 2015; Saunders et al., 2004), defined as distance travelled per unit of energy expended. Because of our evolutionary history, all humans share key anatomical traits that minimise energy expenditure during running, such as long lower limbs, long Achilles tendons, foot arches and short toes (Bramble & Lieberman, 2004; Holowka & Lieberman, 2018; Rolian et al., 2008; Venkadesan et al., 2020). Nevertheless, running economy can vary substantially between individuals and groups (Barnes & Kilding, 2015), particularly between people who run habitually vs. infrequently (Bransford & Howley, 1977; Morgan et al., 1995). Several elements of running technique potentially contribute to this variation, including stride length and rate, leg and thigh kinematics, orientation of the foot at ground strike, ground contact time, vertical oscillation of the centre of mass, torso lean and arm swing (Cavanagh et al., 1977; Folland et al., 2017; Lieberman et al., 2015b; Moore, 2016; Preece et al., 2019; Tartaruga et al., 2012; Trowell et al., 2019; Williams & Cavanagh, 1987; Williams et al., 1987).

Importantly, neither injury risk nor running performance are dictated by any single element of running technique but rather by the combined effects of multiple elements (Folland et al., 2017; Williams & Cavanagh, 1987), as well as other factors such as anthropometry, physical fitness and training, environmental conditions and footwear (Barnes & Kilding, 2015; Cheung & Ngai, 2016; Perl et al., 2012; Raichlen et al., 2011; Saunders et al., 2004; van der Worp et al., 2015). Nevertheless, certain aspects of running technique are more consequential and have received more research attention than others. Recent anthropological research among non-industrial societies has focused on two particular elements of running technique: foot orientation at ground strike and the position of the foot relative to the ipsilateral knee and hip at ground strike (Hatala et al., 2013; Lieberman, 2014; Lieberman et al., 2010, 2015a; Pontzer et al., 2014).

**Figure 1.**
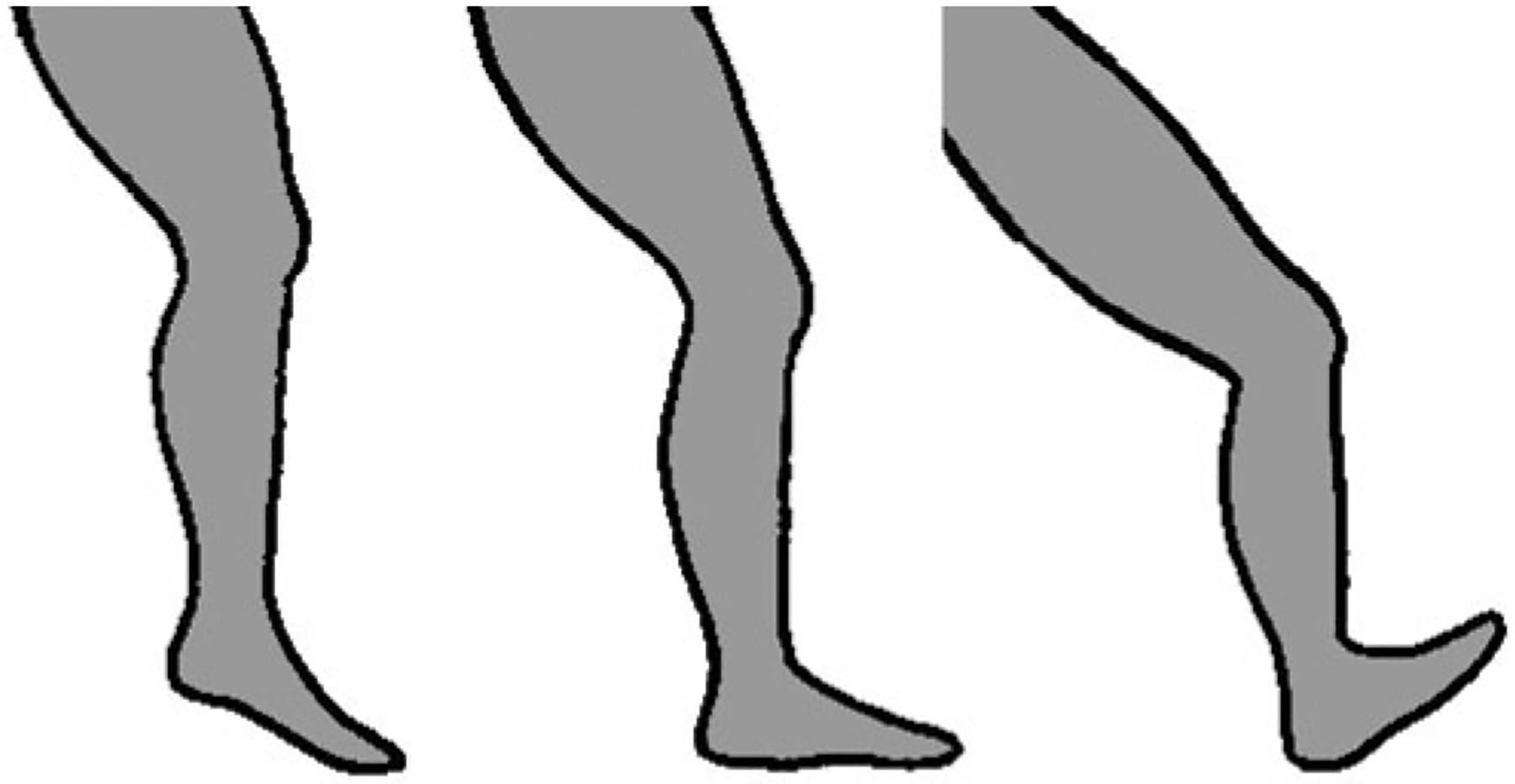
Variation in foot strike patterns during running: forefoot strike (left), midfoot strike (middle), and rearfoot strike (right). Drawing by Samantha Shields.

Orientation of the foot at ground strike is commonly categorised into three types: a forefoot strike, in which the ball of the foot (metatarsal heads) makes contact with the ground first; a midfoot strike, in which the heel and ball of the foot simultaneously contact the ground; and a rearfoot strike, in which the heel first contacts the ground (Figure 1). Evidence indicates that vulnerability to repetitive stress injuries may be greater among runners who rearfoot strike than those who forefoot or midfoot strike (Daoud et al., 2012; Diebal et al., 2012; Roper et al., 2016). One likely cause of this variation in injury risk is the association between different foot strike types and impact transient forces. Experiments have shown that rearfoot striking produces especially large and rapid impact transients, whereas forefoot and midfoot striking typically produce small or no measurable impact transients. The primary reasons for this are that, compared with rearfoot strikes, forefoot and midfoot strikes involve greater ankle compliance and lower effective mass of the decelerating lower limb at ground contact (Lieberman et al., 2010). With regard to performance, whereas most recreational runners tend to rearfoot strike, more top finishers in middle- and long-distance races tend to forefoot or midfoot strike (Hanley et al., 2019; Hasegawa et al., 2007; Hayes & Caplan, 2012; Larson et al., 2011; Preece et al., 2019). It is conceivable that this is because forefoot and midfoot striking provide some advantage in terms of running economy, perhaps by promoting greater elastic energy storage in the Achilles tendons and foot arches, although most experiments designed to test this hypothesis have found running economy to be similar between forefoot, midfoot and rearfoot strikers (Cunningham et al., 2010; Gruber et al., 2013; Perl et al., 2012). Nevertheless, given the potential benefits for injury prevention, and at least no apparent cost for running economy, exercise scientists and coaches often consider forefoot or midfoot striking to be a feature of good running technique (Anderson, 2018; Lieberman, 2021).

**Figure 2.**
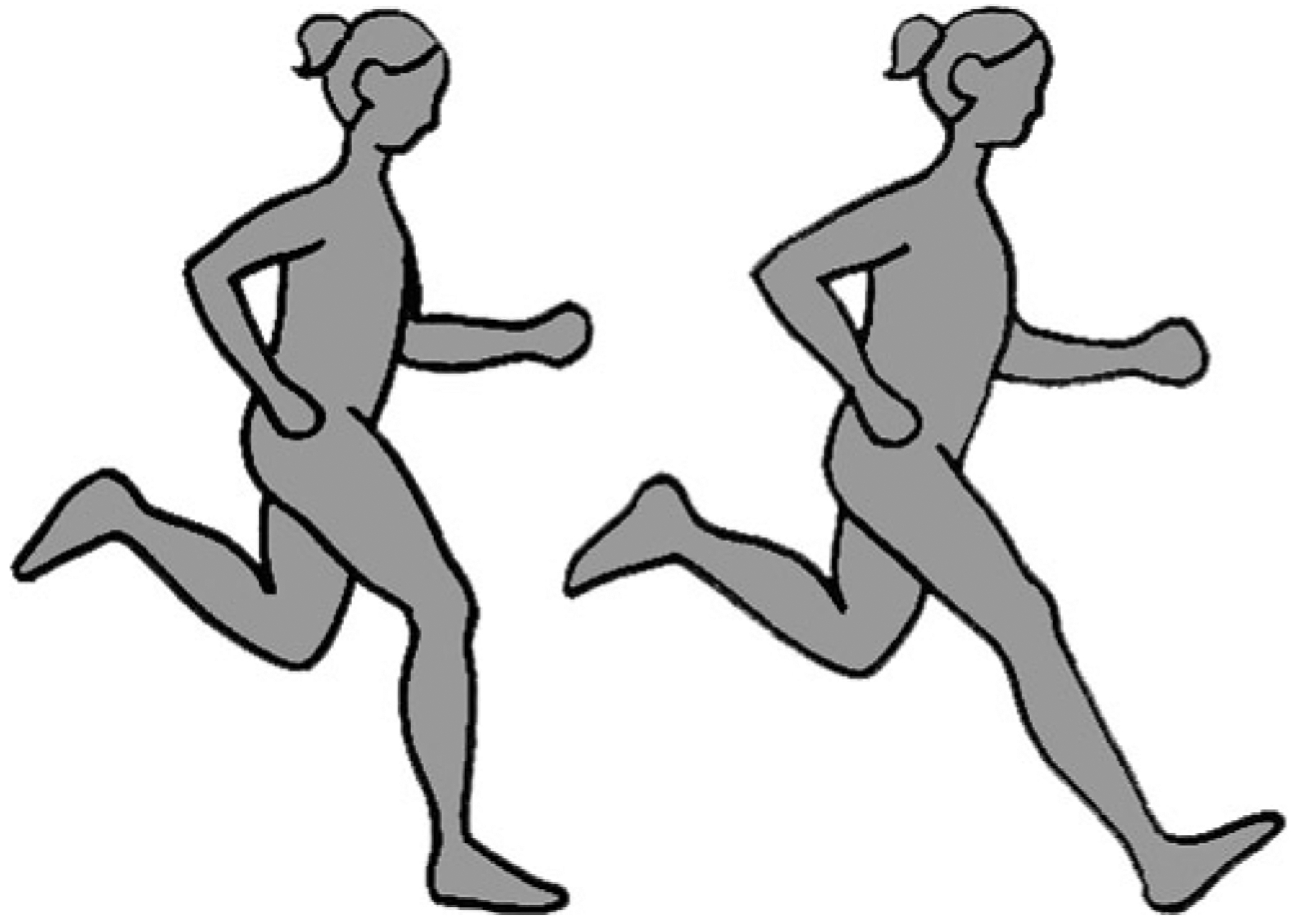
Different orientations of the leg (tibia) at foot strike during running: oriented roughly vertically (left) and at a protracted angle, referred to as overstriding (right). Drawing by Samantha Shields.

The position of a runner’s foot at ground strike relative to their ipsilateral knee and hip is determined by the degree of flexion in their knee and hip. A commonly accepted characteristic of good running technique is landing on the ground with enough knee and hip flexion to position the foot below the knee and orient the leg (tibia) roughly vertically (Anderson, 2018; Daniels, 2014; Lieberman, 2021). Overstriding, defined as contacting the ground with a foot placed far ahead of the knee and hip, is widely considered suboptimal form and occurs when a runner lands with their knee extended and leg oriented at a protracted angle (Figure 2). Studies have shown that, compared with landing with a flexed knee, overstriding produces larger and possibly more rapid impact transient forces (Gerritsen et al., 1995; Lieberman et al., 2015b), primarily because landing with an extended knee increases the stiffness and effective mass of the lower limb (Derrick, 2004). Thus, overstriding is commonly believed to increase risk of injury (Anderson, 2018; Daniels, 2014; Lieberman, 2021). In terms of performance, while overstriding is common among untrained and infrequent runners, more skilled and faster distance runners are often observed to avoid overstriding (Folland et al., 2017; Preece et al., 2019; Trowell et al., 2019), possibly because it negatively affects running economy. In particular, overstriding has been shown to generate especially large braking forces, the posteriorly directed forces in the horizontal plane that slow the body down during running (Heiderscheit et al., 2011; Lieberman et al., 2015b; Napier et al., 2019; Wille et al. 2014). To counter these larger braking forces and maintain a steady running speed, overstriding is expected to require leg and thigh muscles to generate larger forces to propel the body forward, thus costing more energy (Chang & Kram, 1999).

In sum, while recognising that there is no single correct way to run, in terms of the two features of running technique that have recently received research attention from anthropologists, for the purposes of this paper, we define good running technique as involving striking the ground with the forefoot or midfoot and with the leg oriented roughly vertically.

### Running techniques among non-industrial societies

The study of running techniques is a relatively new scientific field that began attracting a large number of researchers only in the 1970s, as a result of the rapid growth of participation in recreational running that occurred in the United States in the late 1960s and early 1970s (Cavanagh, 1990). With this running boom, issues such as injury prevention and performance suddenly became relevant to many more people. Among anthropologists, general interest in running started to grow at roughly the same time, particularly after the publication of a seminal paper by Carrier (1984) which proposed that long-distance running ability represents a key evolutionary adaptation that differentiates humans from other primates. Bramble and Lieberman (2004) later developed this idea more fully, including by demonstrating that fossil evidence for enhanced running ability in the human lineage traces back over a million years. Eventually, interest in running among anthropologists grew to include the topic of running techniques, specifically the body movement patterns that ancient runners might have adopted, millennia prior to the invention of modern running shoes and scientific research on injury risk and performance. This led to a series of studies of the running techniques used by people in contemporary non-industrial societies, with a focus on groups that are habitually barefoot or minimally shod, as people were throughout the vast majority of human evolutionary history.

The first group to be studied were the Kalenjin, an ethnolinguistic group living mainly in the high-lands of western Kenya (Lieberman et al., 2010, 2015a). Traditionally, Kalenjin people subsist on some combination of small-scale farming and pastoralism, and many people are habitually barefoot. The Kalenjin famously include many of the world’s top competitive middle- and long-distance runners (Pitsiladis et al. 2004; Onywera et al. 2006). Running has long been part of Kalenjin culture, owing to the need to run to carry out cattle raids, which is an important custom among some pastoralist groups in East Africa that typically requires running long distances, sometimes well beyond the distance of a marathon (Bale & Sang, 1996; Christensen & Damkjaer, 2010). Western-style track and field sports were first introduced to Kenya by British colonialists during the early twentieth century, and by the mid-1940s, Kalenjin runners were recognised as among the best in the country. By the 1950s, Kalenjin runners were competing internationally, and in the 1960s, Kalenjin runner Kipchoge Keino broke multiple world records, won a gold medal at the 1968 Olympics, and inspired generations of Kalenjins to follow in his footsteps. Since then, numerous other Kalenjin runners have achieved international fame, and running excellence has become a major part of Kalenjin cultural identity. Today, young Kalenjins often grow up running long distances on a regular basis, usually to and from school, many with the hope of one day becoming successful competitive runners (Ojiambo et al., 2012; Onywera et al., 2006).

Lieberman and colleagues (2010, 2015a) studied running techniques among the Kalenjin, focusing on foot orientation at ground strike among rural, habitually barefoot adolescents and adults, and among elite competitive runners who mostly grew up in rural communities and were once habitually barefoot. During barefoot running, both rural individuals and elite runners were observed to mostly land with a forefoot or midfoot strike pattern. Among elite runners wearing cushioned running shoes, forefoot and midfoot striking were still found to be the most common landing patterns, although rearfoot striking was more common than during barefoot running. Based on these findings, Lieberman and colleagues (2010, 2015a) hypothesised that more frequent use of forefoot and midfoot striking used to be greater among runners in the past, before the introduction of modern running shoes.

Additional support for this hypothesis came from a study by Lieberman (2014) of running techniques among the Tarahumara, a Native American group of subsistence farmers living in the Sierra Madre Occidental of northwestern Mexico. For many generations of Tarahumara (who call themselves *Rarámuri*), running has played an integral role in their cultural identity (Lieberman et al., 2020), as it has for other Native American groups in North America (Collier, 2007; Gilbert, 2018; Martin, 2020; Nabokov, 1981). In the past, Tarahumara ran to hunt animals by chasing them over long distances (Levi, 2020; Lieberman et al., 2020; Lumholtz, 1902; Schwatka, 1893), a strategy referred to as persistence hunting (Carrier, 1984), and which has been documented among multiple other non-industrial societies (Estioko-Griffen, 1986; Liebenberg, 2006; Lowie, 1924; McCarthy, 1957; Nabokov, 1981). Today, persistence hunting is rare among the Tarahumara, but many people continue to participate in an ancient custom of long-distance footraces known as *rarajípare* and *ariwete* (Bennett & Zingg, 1935; Irigoyen-Rascón & Palma-Batista, 1994; Lieberman et al., 2020; Pennington, 1963). In *rarajípare*, men run over rugged terrain while kicking and chasing a small wooden ball, often for distances exceeding that of a marathon. In *ariwete*, women run while using a hooked stick to flick a hoop made of stiff plant fibers, usually for distances longer than a half-marathon. These races provide important opportunities to bring together and strengthen ties among communities, and they also serve an important function in Tarahumara spirituality (Lieberman et al., 2020). In recent decades, in addition to engaging in traditional races, many Tarahumara have begun taking part in Western-style long-distance running competitions held in Mexico, and a few top runners have participated and excelled in international competitions (Christensen et al., 2017; McDougall, 2009). For example, among the first Tarahumara runners to gain international recognition were Victoriano

Churro and Juan Herrera who won the Leadville Trail 100 ultramarathon in 1993 and 1994, respectively, while running in traditional Tarahumara attire including minimal sandals consisting only of a piece of stiff material fastened to the bottom of the foot by a leather strap (McDougall, 2009; Severance, 1993). Before roughly a half-century ago, the Tarahumara made the soles of their traditional sandals from animal hides or plant fibers (Bennett & Zingg, 1935; Levi, 1998). Today, however, their sandals’ soles are typically made from the tread surfaces of car tyres (Wallace et al., 2018a), a design innovation that has occurred independently among other non-industrial societies that use minimal footwear (Musiba et al., 1997; Somjee, 1993).

**Figure 3.**
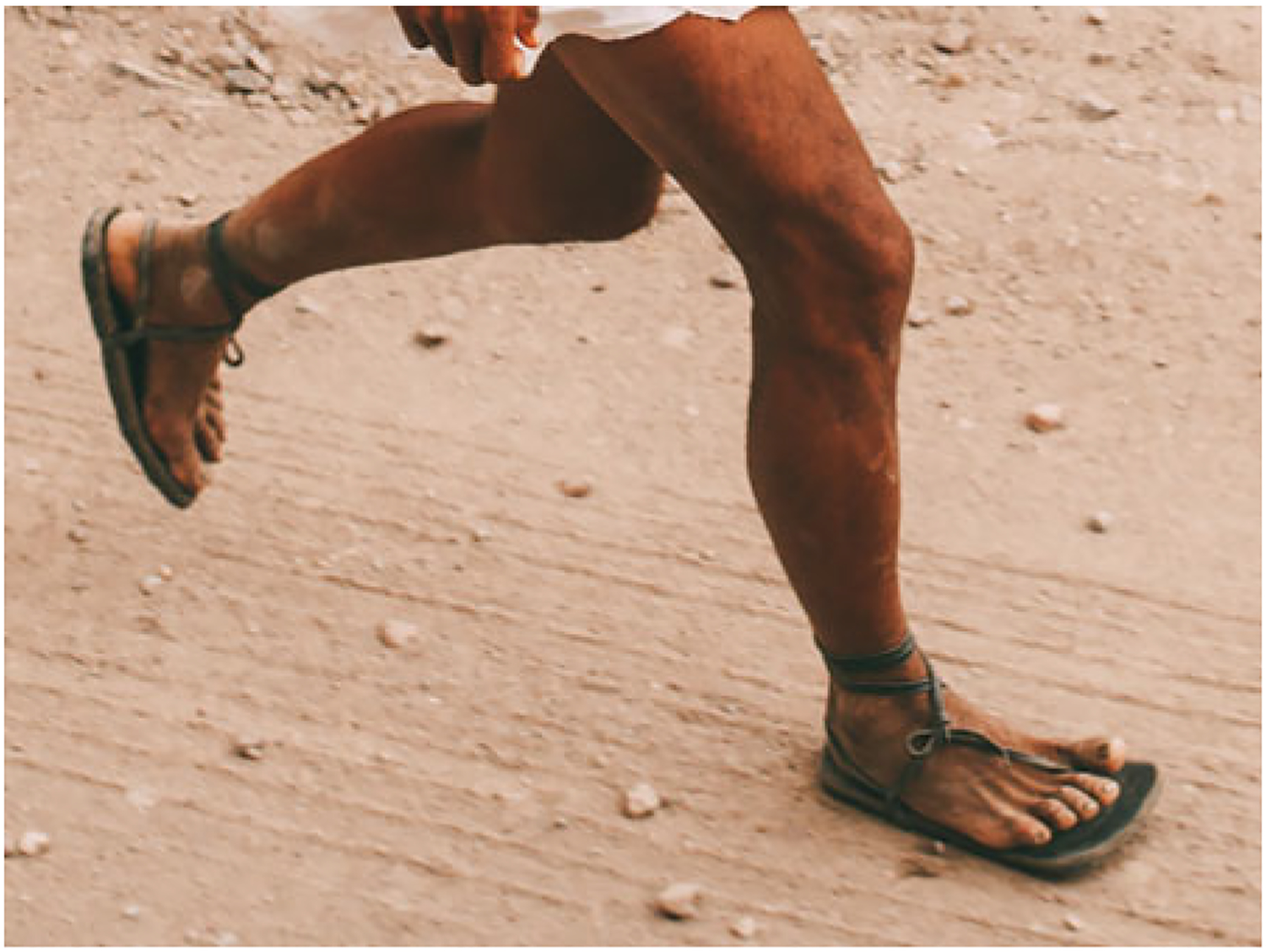
Tarahumara runner in Mexico landing with a midfoot strike pattern and leg that is oriented roughly vertically. Photo by David Ramos and used here with permission.

Lieberman’s (2014) study of running techniques among the Tarahumara focused on foot strike patterns and lower limb kinematics in a sample of Tarahumara farmers who habitually wore traditional sandals, all of whom were experienced *rarajípare* runners and a few of whom had competed in national and international ultramarathons. It was observed that, while running in their minimal sandals, the Tarahumara rarely landed with a rearfoot strike pattern. Also, overstriding was rare. Instead, Tarahumara runners tended to use either a forefoot or midfoot strike pattern and land with their legs oriented roughly vertically (Figure 3). Therefore, consistent with Kalenjin runners, Tarahumara runners were found to adopt key features of what many exercise scientists and coaches consider to be good running technique.

However, studies of running techniques among two other non-industrial societies – the Daasanach and Hadza – showed different patterns than those reported for the Kalenjin and Tarahumara (Hatala et al., 2013; Pontzer et al., 2014). The Daasanach, who live primarily in southern Ethiopia and northern Kenya near the Omo River and Lake Turkana, are traditionally pastoralists, although many people today also subsist on small-scale farming and fishing. The Hadza live in northern Tanzania near Lake Eyasi and are traditionally hunter–gatherers. Among both the Daasanach and Hadza, nearly all people are habitually barefoot or wear minimal sandals made from car tyre treads (Musiba et al., 1997), similar to Tarahumara sandals. Hatala and colleagues (2013) examined foot strike patterns during barefoot running among a sample of Daasanach adults in northern Kenya and found that rearfoot striking was very common, midfoot striking was much less common and forefoot striking was very rare. Pontzer and colleagues (2014) examined foot strike patterns among a sample of Hadza juveniles and adults running barefoot and in minimal sandals and found that, both with and without footwear, rearfoot and midfoot striking were roughly equally common and forefoot striking was never observed. Overstriding was not quantified in either study, but figures in both the paper by Hatala et al. (2013) and Pontzer et al. (2014) illustrating typical Daasanach and Hadza rearfoot strikes, respectively, clearly depict people who are overstriding. Considered together with the results from the Kalenjin and Tarahumara, these findings in just four populations demonstrate that running techniques are variable among people in contemporary non-industrial societies, and hence also probably were among people living in the ancient past. Yet, if simply being barefoot or minimally shod does not dictate running techniques among non-industrial societies, what factors are important?

One possible explanation for why running techniques among the Daasanach and Hadza were found to differ from those of the Kalenjin and Tarahumara is that unlike among the Kalenjin and Tarahumara, running is not a particularly common activity among the Daasanach or Hadza, nor is there any indication that it is an important part of cultural identity. In other words, among non-industrial societies, running techniques potentially vary between groups according to the cultural importance of running. Elements of what is considered good running technique may be more likely to be developed among groups in which people run on a regular basis and consider running to be an important part of their culture, whereas suboptimal running technique may be more common among groups in which running is not considered culturally important and there are few opportunities to develop elements of good running technique. If so, variation in running techniques among non-industrial societies may represent an elegant and underappreciated example of how culture influences how people use their bodies to move.

To further explore this hypothesis, we studied running techniques among the Tsimane, a forager–horticulturalist group living in the Bolivian Amazon. We also objectively measured the frequency of running among the Tsimane, and as we show below, running was not found to be a common activity. Therefore, we hypothesised that, similar to the Daasanach and Hadza, Tsimane would commonly adopt elements of suboptimal running technique, specifically rearfoot striking and overstriding.

## Methods

### The Tsimane

The Tsimane are an indigenous group living in the tropical lowland of the Bolivian Amazon (Beni Department). They subsist on a combination of slash-and-burn horticulture (primarily plantains, rice, manioc and maize); fishing in rivers, streams and lagoons; hunting a variety of neotropical mammals; and gathering of fruits, nuts, honey and other foods. The Tsimane inhabit over 90 villages of approximately 50–500 people located along rivers and interfluvial terra firma. Since the early 2000s, numerous aspects of Tsimane behaviour, culture, and biology have been studied as part of the ongoing longitudinal research programme known as the Tsimane Health and Life History Project (Gurven et al., 2017). This research has shown, among other things, that the Tsimane tend to be very physically fit compared with many people in post-industrial societies, including by having excellent aerobic and cardiovascular fitness into older age (Kaplan et al., 2017; Pisor et al., 2013). Tsimane people also engage in high levels of physical activity across their lifecourse, primarily for purposes of subsistence (Davis & Cashdan, 2019; Gurven et al., 2013; Kraft et al., 2021; Stieglitz et al., 2017). For example, Tsimane women and men walk, on average, 7.6 and 9.9 km per day, respectively (Davis et al., 2022).

Nevertheless, throughout roughly two decades of research among the Tsimane, there has been no indication that running is an especially common activity or that running is an important part of Tsimane cultural identity. Hunting practices, for example, have been studied extensively among the Tsimane (Gurven et al., 2006; Hooper et al., 2015; Schniter et al., 2015; Trumble et al., 2014), yet there has never been an observation or report of persistence hunting taking place, either recently or in the past. Tsimane hunters often sprint for brief periods to chase or outmanoeuvre prey animals, and to follow hunting dogs when in pursuit, but they seemingly never utilise endurance running to exhaust their prey. In addition, there has never been an observation or report of customary footraces taking place among the Tsimane. In recent years, football (soccer) has become a popular recreational activity among some Tsimane, which involves running intermittently at submaximal speeds, but the Tsimane seemingly do not engage in sports that require sustained running at aerobic intensity over long distances. Anecdotes aside, however, running has never been formally investigated among the Tsimane, hence the motivation for this study.

Another topic for which data are currently lacking is the footwear habits of the Tsimane. Our impression from fieldwork has been that the majority of Tsimane are habitually barefoot, especially during childhood and adolescence, but this has never been quantified. Among people who wear shoes, traditionally the most common types of footwear are minimal sandals made from car tyre treads (called *abarca* by the Tsimane). However, these have been increasingly replaced by commercially available flip-flop sandals (*chinelas*). Football cleats are now often worn by men, but only during recreational football matches. Given anthropological interest in relationships between footwear and running techniques among non-industrial societies, for this study, in addition to assessing running frequency and techniques, we also evaluated patterns of footwear use among the Tsimane.

### Footwear frequency

To assess footwear use patterns among the Tsimane, a sample of 2,099 females and 1,992 males were surveyed in 83 villages. These data were collected primarily for the purposes of a separate study focused on the influence of footwear on risk of hookworm infection through the feet. Participants ranged in age from young children to elderly adults. Participants were asked whether they never wear shoes, rarely wear shoes, sometimes wear shoes, usually wear shoes or always wear shoes. For young children, responses were often obtained from a parent.

### Running frequency

To assess the frequency of running among the Tsimane, 43 women and 30 men were recruited from four villages. Participant movement was recorded using GPS data loggers (QStarz BT-Q1000XT, Qstarz Int. Co., Taipei, Taiwan) worn around the neck on a lanyard. Movement was recorded every 10 seconds – which is considered by the manufacturer to be optimal for recording travel on foot while conserving battery power – over three consecutive days per participant. Power limitations in remote areas and technical difficulties owing to inclement weather resulted in some missing data (13.7% of participants had only two days with recorded tracks, rather than three). After GPS data were collected, GPS tracks were cleaned of spurious points by the DIGIT Lab at the University of Utah. To determine time spent running, first, we calculated travel speeds for all GPS point intervals by dividing distance moved by interval time. Second, because a few participants occasionally travelled by motorbike or taxi while wearing GPS units, we developed a custom algorithm to filter out intervals that involved motorised travel. In brief, we identified any intervals with travel speeds greater than 6.5 m/s and removed these points along with 100 surrounding consecutive points (corresponding to approximately 15 minutes) on either side within a given person-day. This procedure was effective at eliminating cases where acceleration or slow movement on roads produced plausible running speeds but manual investigation of GPS tracks indicated clear motorised transit. Third, we filtered out any remaining points where people travelled greater than 5 m/s, which we considered to be an upper-bound of realistic possible running speeds. Fourth, we converted travel speeds to dimensionless speeds (Froude number) as:

$${\text{Dimensionless speed = (travel speed)}}^{2}/(\text{hip height }\times g).$$

where travel speed is in m/s (over epochs with median duration of 10 seconds), hip height (in metres) is calculated from standing and sitting heights and *g* is the gravitational constant (9.81 m/s^2^). Fifth, following Pontzer and colleagues (2014), we calculated the percentage of movement bouts that indicated running by removing all intervals with travel speeds <0.5 m/s and dividing the number of bouts with dimensionless speeds >0.5 (the threshold between walking and running; Alexander & Jayes, 1983) by the total number of bouts.

### Running kinematics

To assess running techniques among the Tsimane, a group of 17 women and 15 men were recruited from the village of Jämsi Bayedye. Average (SD) stature was 152.4 (4.2) and 163.8 (8.1) cm among female and male participants, respectively. Average body weight was 59.1 (7.4) and 63.9 (8.9) kg among females and males, respectively. No participants reported any injury that would affect running ability, and none exhibited any visible problems with their gait. Among participants, 88% reported having been habitually barefoot for much of their lives and 34% reported having been habitually barefoot for their entire lives. All participants reported that they rarely run except for brief periods, for example, to escape danger, sprint after animals during hunting and while playing football.

Participants were recorded with a video camera (Casio EX-ZR100, Casio Computer Co., Tokyo, Japan) while running over the two types of terrain that Tsimane people most often encounter: an open, flat ground surface of hard-packed dirt located in the village, and a relatively flat dirt trail through forest surrounding the village. In both settings, evenly spaced flags were used to mark the running path. Participants were instructed to run at a speed that they would be comfortable running at for several minutes. They were told to maintain this speed and not decelerate until they had passed a flag approximately 3 m beyond the camera. The camera was positioned perpendicular to the running routes at a height of approximately 75 cm and distance between 3 and 5 m. The capture rate of the camera was 240 frames per second. Two trials were recorded for each participant in each setting. During all trials, participants ran barefoot.

Prior to recording running trials, reflective tape markers were affixed to the following anatomical locations on the right sides of participants’ bodies: greater trochanter (hip), centre of the knee (between the lateral femoral epicondyle and lateral tibial plateau), lateral malleolus and the lateral surface of the fifth metatarsal head. All anatomical locations were determined by manual palpation. The greater trochanter marker was affixed to the participant’s overlying clothes, but all other markers were affixed directly on the participant’s skin. Lower extremity joint angles were measured at the moment of foot strike, defined as the first video frame in which the right foot made contact with the ground. Foot strike angle was measured as the angle between the ground plane and a line connecting the heel surface and distal fifth metatarsal. Positive angles correspond to rearfoot striking, negative angles correspond to forefoot striking, and an angle of zero corresponds to midfoot striking. Ankle angle was measured as the angle between the lines from the knee centre to the lateral malleolus and from the lateral malleolus to the distal fifth metatarsal. Overstride was defined as how far the ankle landed anterior to the knee and was measured as the angle between the leg (from the lateral malleolus to the knee centre) and a vertical line between the knee centre and ground plane. An angle of zero or less indicates no overstride, and higher angles indicate more overstride. An angular measurement of overstride was used rather than a linear measurement because there is less error in measuring the angle of the leg than the projected distance between the knee centre and foot (Lieberman, 2014). Stride length was measured as the distance between two consecutive foot strikes, and dimensionless stride length was calculated by dividing stride length by lower extremity length (greater trochanter height) to standardise for body size variation among participants. Running speed was measured based on the horizontal translation of the tape marker placed on the greater trochanter, from which dimensionless speed was calculated to standardise for body size variation. All camera data were analysed using ImageJ software (v. 1.50i, NIH, Bethesda, MD, USA). Trials were discarded when body markers were obscured in the camera recordings. The final sample was 78 trials (*n* = 52 in the open setting).

To assess sources of variation in lower extremity kinematics during running, we used general linear mixed models (GLMMs). In a main set of analyses, joint angles and dimensionless stride length were included as dependent variables, dimensionless speed, setting (open vs. forest) and sex were included as fixed effects, and participant identity was included as a random effect. A separate GLMM was used to assess the association between foot strike angle and overstride angle, which included foot strike angle as the dependent variable, overstride angle, dimensionless speed, setting and sex as fixed effects, and participant identity as a random effect. Another GLMM was used to assess the association between dimensionless speed and setting, which included dimensionless speed as the dependent variable, setting and sex as fixed effects, and participant identity as a random effect. All statistical analyses of kinematic data were conducted using JMP Pro software (v. 16.0, SAS Institute, Cary, NC, USA), with statistical significance set at *p* < 0.05.

## Results

**Figure 4.**
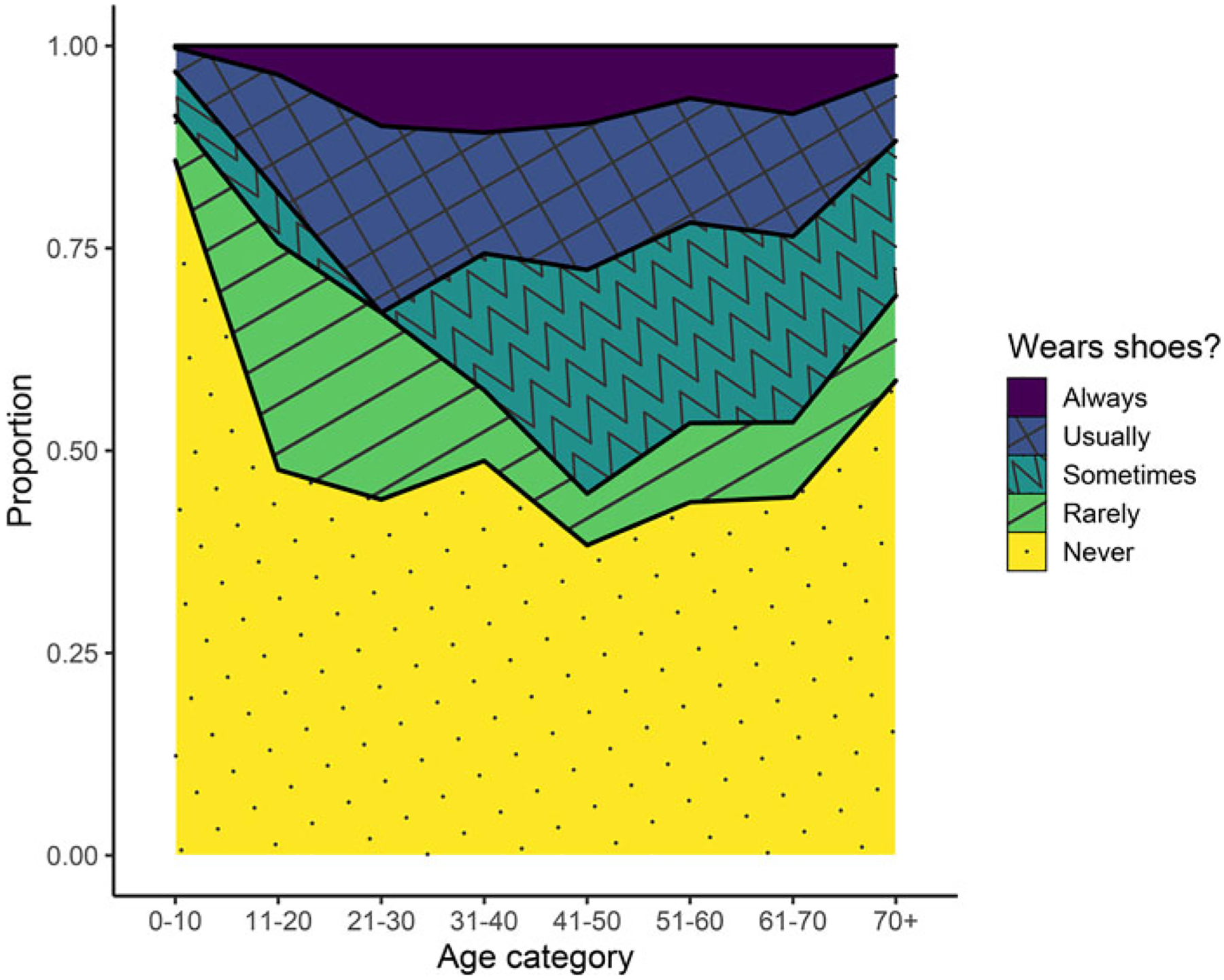
Footwear habits among the Tsimane across different age categories.

Among the Tsimane surveyed about their footwear habits, 65% of all people reported never or rarely wearing shoes (Figure 4). Always being barefoot or rarely wearing shoes was especially common among children and adolescents, but even among adults, a minimum of approximately 50% of people at all ages reported never or rarely wearing shoes. Among all age groups, less than 15% of people reported always wearing shoes.

**Figure 5.**
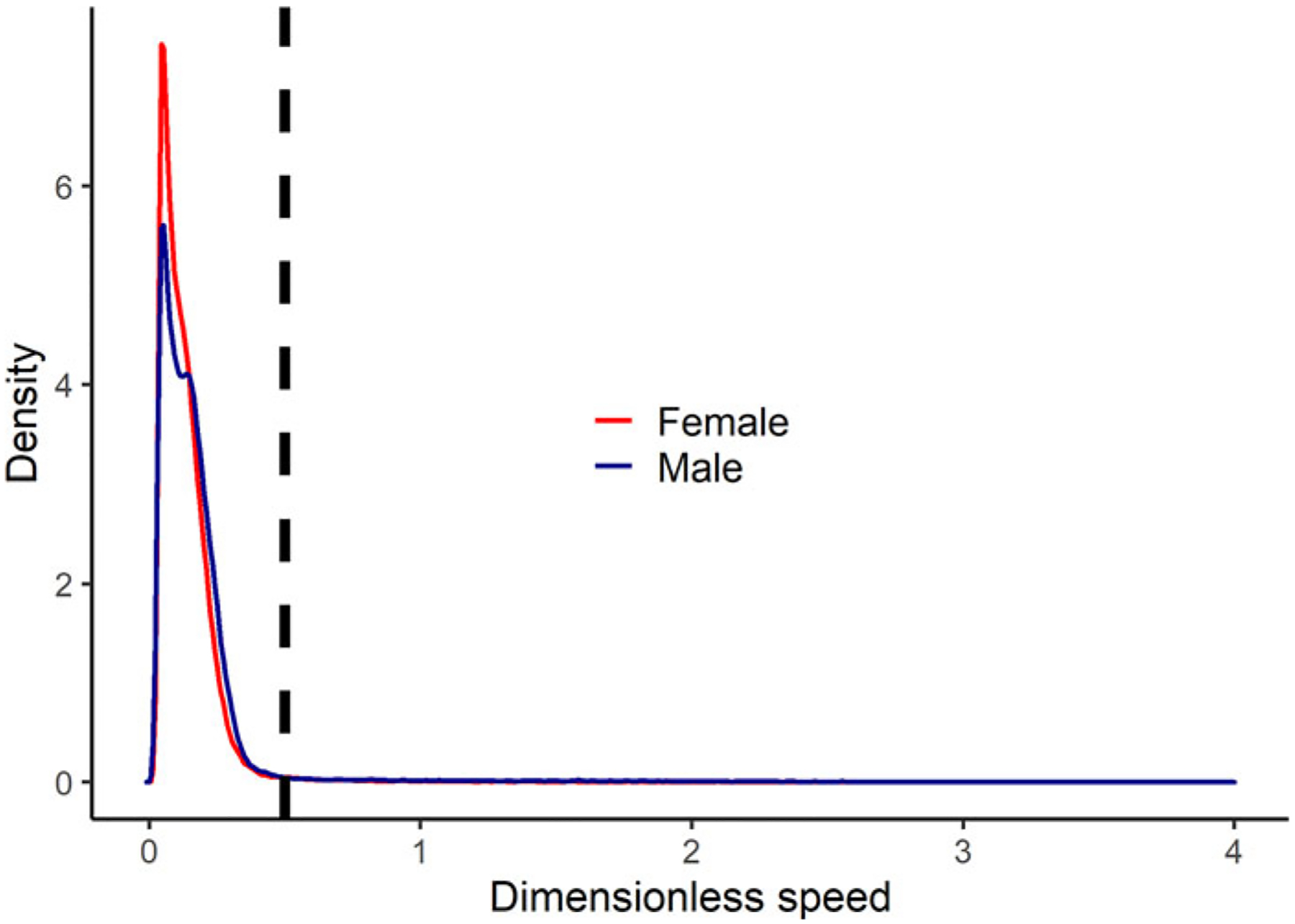
Kernel density plot of dimensionless speed (Froude number) by sex among the Tsimane who wore GPS units to measure their physical activity patterns. The dashed line indicates the threshold above which dimensionless speed represents running (dimensionless speed >0.5). The plot only includes recorded bouts in which GPS recordings indicated that participants were actively moving (travel speeds >0.5 m/s).

Among the Tsimane adults who wore GPS units to measure their movement patterns, running was very rare among both women and men (Figure 5). Across all timepoints during which participants were recorded to be actively moving, only 2.4% of bouts occurred at dimensionless speeds that indicated running (Froude number >0.5).

**Figure 6.**
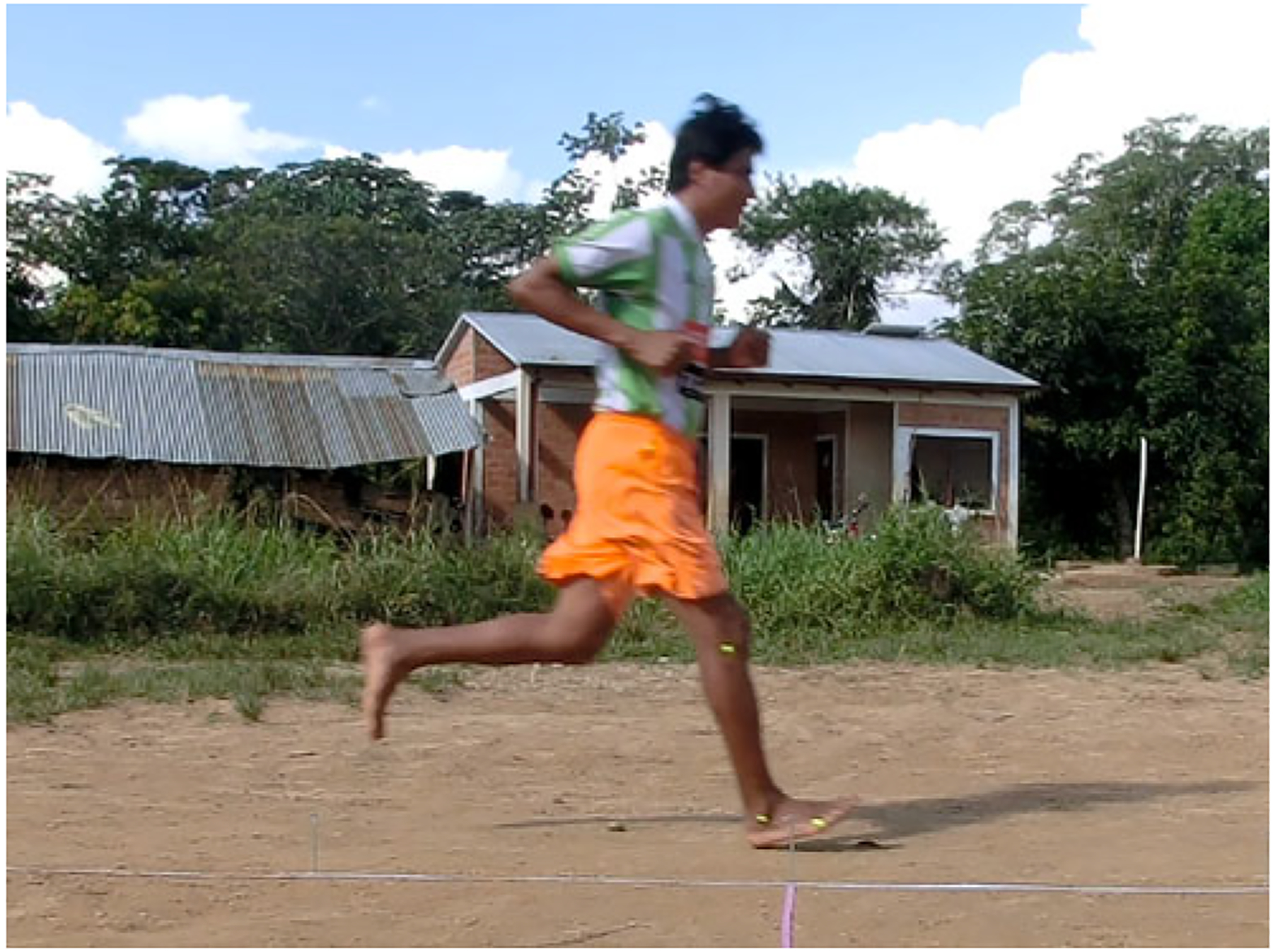
Tsimane study participant in Bolivia running with a rearfoot strike pattern and overstriding.

**Figure 7.**
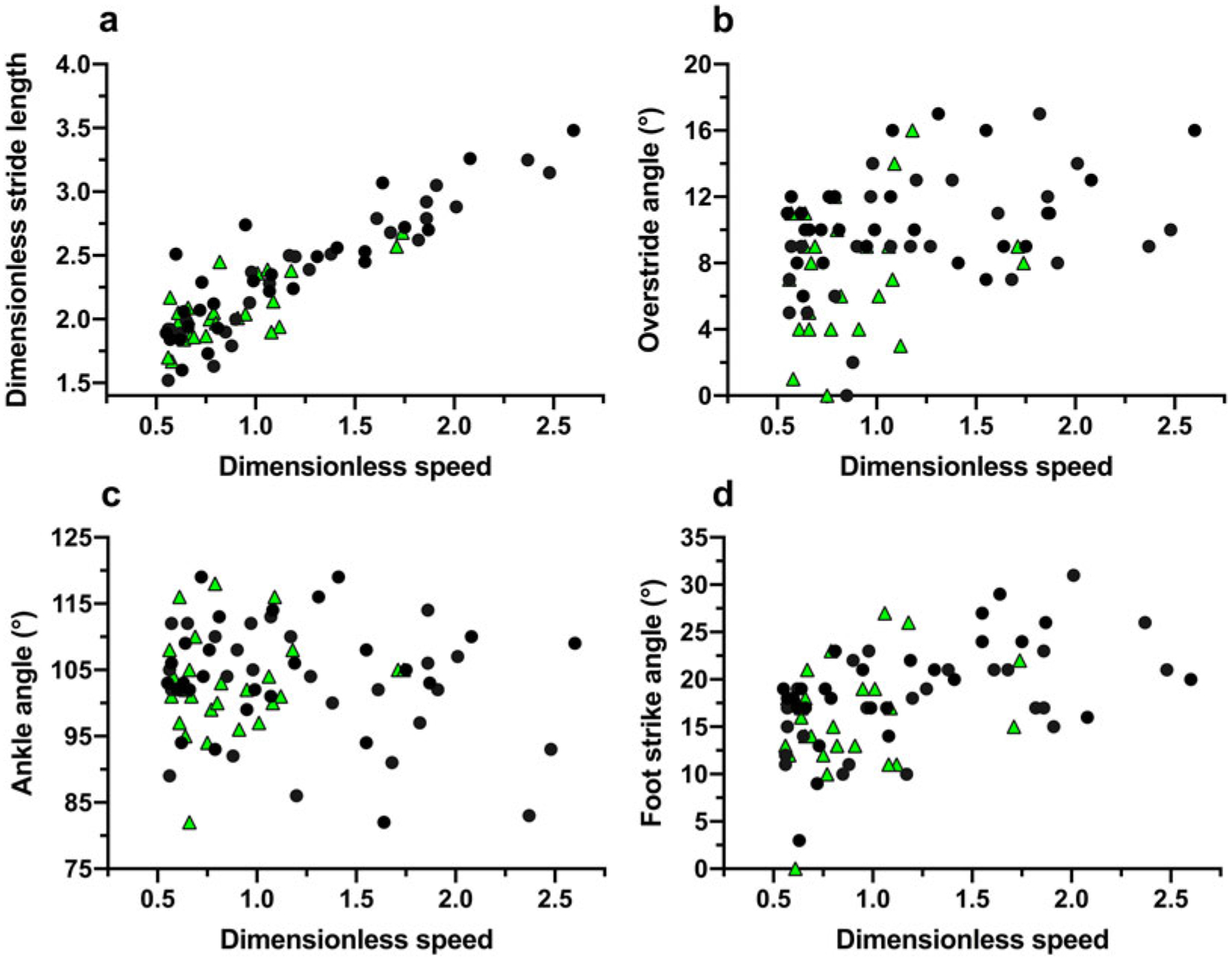
Running kinematic variables analysed among the Tsimane relative to dimensionless speed (Froude number): dimensionless stride length (a), overstride angle (b), ankle angle (c), and foot strike angle (d). Black circles indicate open setting trials and green triangles indicate forest trail trials.

**Figure 8.**
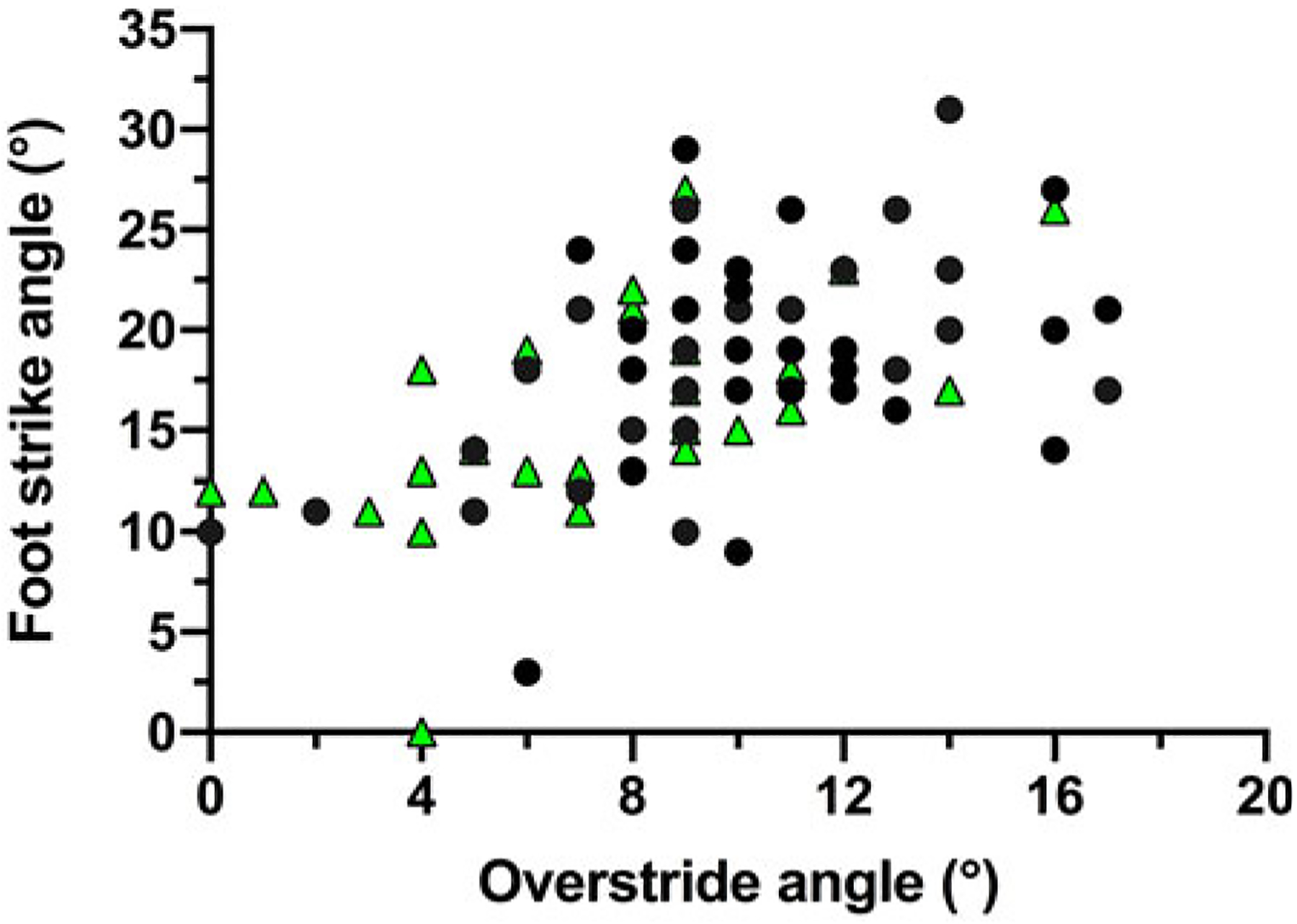
Association between foot strike and overstride angles among the Tsimane during running. Black circles indicate open setting trials and green triangles indicate forest trail trials.

In trials to assess running techniques among a sample of Tsimane adults, rearfoot striking and overstriding were both very common (Figures 6 and 7). No participant was ever observed to forefoot strike, midfoot striking was observed only once and rearfoot striking was observed in 99% of trials. In only two trials did participants land with their legs oriented vertically, whereas overstriding occurred in 98% of trials. Foot strike and overstride angles were positivity associated (*p* = 0.0043), such that foot strike angles were greater when participants overstrode more (Figure 8). Foot strike and overstride angles both increased at higher dimensionless speeds (*p* < 0.0001 and *p* = 0.0021, respectively), as did dimensionless stride lengths (*p* < 0.0001). Ankle angles, however, were not significantly affected by dimensionless speed (*p* = 0.43). In the forest, dimensionless speeds were slower than in the open setting (*p* = 0.011). After controlling for this variation in dimensionless speed, overstride angles and dimensionless stride lengths were lower in the forest than the open setting (*p* = 0.018 and *p* = 0.038, respectively), while foot strike and ankle angles were not significantly affected by setting (*p* = 0.25 and *p* = 0.49, respectively). The only kinematic variable for which sex was a significant predictor was overstride angle, which tended to be greater in females than males (*p* = 0.038).

## Discussion

Our findings from research among the Tsimane add to evidence from other non-industrial societies that body movement patterns adopted during running tend to vary between groups according to the cultural importance of running. Similar to the Daasanach and Hadza, Tsimane people rarely run, and there are no indications that running is considered an important part of their cultural identity. Also similar to the Daasanach and Hadza, when Tsimane people do run, they tend to adopt elements of what many exercise scientists and coaches consider to be suboptimal running technique, specifically rearfoot striking and overstriding. In these ways, the Tsimane are different from the Kalenjin and Tarahumara, for whom running is a common activity and a major part of cultural identity, and who tend to use elements of what is considered good running technique for preventing injury and maximising performance, specifically forefoot or midfoot striking and landing with a roughly vertically oriented leg. Although the processes by which culture influences variation in running techniques among non-industrial societies are not currently known, we propose two possibilities that warrant future investigation.

First, among non-industrial societies in which running is a routine activity, people may be more likely to adopt elements of good running technique because they have had more opportunities to self-optimise their movement patterns through trial and error. This kind of fine-tuning of a person’s running technique could be entirely subconscious and not require any social learning. Ample evidence of self-optimisation has been provided by experiments with runners in laboratory settings. For example, a common experimental design has been to measure a person’s preferred stride length and frequency as they run on a treadmill, and then systematically vary (increase and decease) their stride length and frequency while simultaneously measuring energy expenditure in order to determine their energetically optimal stride length–frequency combination. In such experiments, frequent runners are consistently observed to naturally choose stride length–frequency combinations that closely match their experimentally determined most economical stride lengths and frequencies (Cavanagh & Williams, 1982; Hunter & Smith, 2007; Connick & Li, 2014), whereas inexperienced runners tend to choose less economical stride length–frequency combinations (de Ruiter et al., 2014; van Oeveren et al., 2017). In all likelihood, many other elements of running technique are, at least in part, shaped by self-optimisation, including lower extremity kinematics (Moore, 2016; Moore et al., 2012, 2019; Williams & Cavanagh, 1987). Moreover, owing to the heightened somatosensory feedback associated with being barefoot or minimally shod, self-optimisation might be expected to have an especially strong influence on running technique development among many non-industrial societies (Lieberman, 2012).

Second, in addition to the effects of self-optimisation, among non-industrial societies in which running is common and an important part of cultural identity, people may tend to develop elements of good running technique as a result of social learning. Most likely, this would occur primarily through observational and imitative learning rather than explicit instruction. Numerous intervention studies with runners in post-industrial societies have shown that many elements of running technique can be altered through training and coaching, including lower extremity kinematics (Bailey & Messier, 1991; Clansey et al., 2014; Craighead et al., 2014; Crowell & Davis, 2011; Dallam et al., 2005; Diebal et al., 2012; Fletcher et al., 2008; Messier & Cirillo, 1989; Morgan et al., 1994). Among non-industrial societies, however, we are not aware of any documented cases of deliberate teaching of running technique. Competitive Kalenjin runners often receive substantial coaching, but not until they have already achieved elite status and their running technique has largely been established. Also, the coaching is often from non-Kalenjins (Wilber & Pitsiladis, 2012). Nevertheless, people in every society develop important skills by watching and imitating others who are good at them (Henrich, 2016; Henrich & Broesch, 2011), and there is no reason that this would not be the case for running. Among non-industrial societies, customary public displays of running such as the traditional *rarajípare* and *ariwete* footraces of the Tarahumara would provide especially useful opportunities for observational learning. Indeed, Tarahumara runners often say when asked how they learn to run that they follow the techniques of champion footracers (Lieberman, 2021). Persistence hunting, cattle raiding and any other activity in which people might run in a group could also provide valuable chances to watch and learn the techniques of skilled runners.

Footwear is another dimension of culture that may have some influence on running techniques among non-industrial societies, but not necessarily in a straightforward way. Studies of habitual runners in post-industrial societies have often found that people who run barefoot typically forefoot or midfoot strike and take shorter strides compared with shod runners, leading to less overstride (DeWit et al., 2000; Divert et al., 2008; Hamill et al., 2011; Kerrigan et al., 2009; Larsen, 2014; Lieberman et al., 2010; Squadrone & Gallozi, 2009). However, previous data from the Daasanach and Hadza (Hatala et al., 2013; Pontzer et al., 2014), and new data presented here from the Tsimane, indicate that plenty of habitually barefoot people in some non-industrial societies run with a rearfoot strike pattern and overstride. Thus, cultural footwear norms alone do not determine good running technique. Nevertheless, it remains very possible that were Daasanach, Hadza and Tsimane people to run more frequently, doing so barefoot as opposed to shod would more strongly encourage them to adopt forefoot or midfoot striking and avoid overstriding. In other words, there are likely to be interaction effects on running techniques between footwear patterns and frequency of running.

Without a doubt, cultural factors are not the only determinants of running techniques in non-industrial societies. Among other potential influences, variation in local environmental conditions probably plays a major role. Experiments in laboratory settings have shown that runners vary their body kinematics when running on different surfaces, including flat vs. sloped surfaces (Gottschall & Kram, 2005; Lussiana et al., 2013; Padulo et al., 2012; Swanson & Caldwell, 2000), even vs. uneven surfaces (Dhawale & Venkadesan, 2021; Voloshina & Ferris, 2015) and stiff vs. compliant surfaces (Ferris et al., 1998; Pinnington et al., 2005; Tung et al., 2014). Natural terrains clearly also vary in terms of slope, evenness and stiffness, making it probable that these and other surface characteristics contribute to shaping running techniques among non-industrial societies. In this study, we found running kinematics among the Tsimane to differ when participants ran on a forest trail vs. in an open setting. In both settings, the running surfaces were relatively flat and consisted of hard-packed dirt. Yet, on the forest trail, participants ran slower with shorter stride lengths and less overstride. Our suspicion is that these differences in running kinematics between forest and open settings are prompted primarily by safety considerations rather than differences in surface characteristics, as previous laboratory studies found little effect of surface irregularity or compliance on stride length (Ferris et al., 1998; Voloshina & Ferris, 2015). Forests are cluttered with potential obstacles like fallen branches and roots that may occur on trails, which could lead people to constrain stride length and proceed more slowly and cautiously during running. This, in addition to the fact that animals in forests can easily hide and cannot be seen over long distances, could preclude persistence hunting from being a viable option in certain forest environments (but see Estioko-Griffen, 1986). Ultimately, more research is needed to better understand the ways in which environmental variation influences running techniques among non-industrial societies.

Throughout this paper, it has been implied that suboptimal running technique is a kind of default strategy that people are expected to adopt unless they learn to do otherwise. Although this is a hypothesis that remains to be tested, we consider it a reasonable assumption. Specifically, we suspect that among people who rarely run, it feels most natural to use kinematic patterns that are similar to those employed during the much more common activity of walking. During walking, nearly all people in every society, regardless of footwear, usually make initial contact with the ground with their rearfoot and have a roughly fully extended knee (Addison & Lieberman, 2015; Alexander, 1992; Holowka et al., 2019; Muybridge, 1887; Wallace et al., 2018a; Willems et al., 2017), including the Tsimane (Holowka et al., in press). In part, this is likely because during walking, landing on the rearfoot provides a clear benefit in terms of energy savings (Cunningham et al., 2010). If running is an unfamiliar activity, it would make sense that a person would resort to an established motor programme for a familiar activity such as walking, and as a result be prone to rearfoot striking and overstriding. Moreover, when walking at faster speeds, people almost always take longer strides (Bertram & Ruina, 2001), so there may be a natural tendency to transition into running by taking even longer strides, which would also increase the likelihood of rearfoot striking and overstriding. A natural tendency to rearfoot strike may partly explain why even some elite runners have been observed to habitually use a rearfoot striking pattern (Hanley et al., 2019; Hasegawa et al., 2007; Hayes & Caplan, 2012). Among these runners, learned adjustments to other aspects of their technique could compensate for any negative consequences of rearfoot striking for injury risk and performance (Williams & Cavanagh, 1987).

When considering variation in running techniques, it is important to keep in mind that all humans possess numerous anatomical and physiological adaptations that make our species better at running long distances compared with other primates. These adaptations almost certainly originated long ago in the human evolutionary lineage, very likely among early members of *Homo* during the Early Pleistocene (Bramble & Lieberman, 2004). Examples of such adaptations can be found across diverse systems in our bodies including the musculoskeletal system (Bramble & Lieberman, 2004; Eng et al., 2015; Holowka & Lieberman, 2018; Lieberman et al., 2006; Rolian et al., 2008; Venkadesan et al., 2020), cardiopulmonary system (Bramble & Carrier, 1983; Callison et al., 2019; Shave et al., 2019), thermoregulatory system (Carrier, 1984; Kamberov et al., 2018; Lieberman, 2015) and nervous system (Raichlen et al., 2012; Wallace et al., 2018b). Thus, from a broad evolutionary perspective, regardless of variation in running techniques, humans are in general ‘good’ runners, or at least we have an evolved natural capacity to run well. Nonetheless, a key takeaway from this study is that even with this species-wide natural capacity, running is still a skill that requires learning and practice for it to be fully mastered. This was presumably just as true among our ancient ancestors as it is for people today. Unfortunately, the degree to which, and ways in which, running techniques varied among ancient cultures are impossible to know. Even so, we suspect that by at least the Middle Pleistocene (probably earlier), as our ancestors were occupying increasingly diverse environments, the efficacy of persistence hunting would have varied among groups in different habitats. This alone would probably have resulted in running being a more common and important activity among some groups than others, which we would expect to give rise to variation in running techniques. Social learning might have been an important process by which ancient runners developed their techniques, similar to how social learning has been hypothesised to have been critical to the development of Paleolithic stone tool manufacturing techniques (e.g. Pargeter et al., 2019; Stout et al., 2019; Tostevin, 2012).

In conclusion, much remains to be learned about how culture influences the body techniques employed during running among non-industrial societies, as well as the techniques used in many other types of physical activity. Admittedly, our research with the Tsimane was not fully comprehensive. The kinematic variables analysed were limited to those examined in recent anthropological studies of other non-industrial societies, but more variables could have been considered. Also, kinetic variables were not considered at all. Although good evidence was provided that running is an uncommon activity among the Tsimane, our conclusion that running is not a major part of Tsimane cultural identity was based on informal impressions rather than systematic interviews. Furthermore, we did not investigate the roles that self-optimisation, social learning or other processes might play in the development of Tsimane running techniques. Hopefully, future studies will take a more thorough and multidisciplinary approach to analysing running techniques among non-industrial societies. Nevertheless, we hope that the ideas and evidence presented here will help motivate such future studies. Beyond being of interest from an anthropological perspective, achieving a better understanding of how culture influences running techniques among non-industrial societies would provide exercise scientists, coaches and the general public with a more holistic understanding of the factors influencing running performance and injury risk, as well as a more nuanced view of how humans evolved to run.

## Supplementary Material

1

## Data Availability

Data from the Tsimane who participated in the running kinematics trials are available in the Supplementary Material. Data on Tsimane footwear frequency and GPS data used to determine running frequency among the Tsimane are available on reasonable request from MG and HED, respectfully.
